# Finite element analysis of plate placement in canine elbow arthrodesis: comparison of caudal, medial, and lateral applications

**DOI:** 10.3389/fvets.2025.1705303

**Published:** 2026-01-16

**Authors:** Junggyu Shin, Jongchan Ko, Suyoung Heo

**Affiliations:** Department of Veterinary Surgery, College of Veterinary Medicine, Jeonbuk National University, Iksan, Republic of Korea

**Keywords:** biomechanical test, custom plate, dogs, elbow arthrodesis, finite element analysis

## Abstract

**Introduction:**

Canine elbow arthrodesis is a salvage procedure that reduces pain while preserving minimal limb function. Historically, plates have been applied to the caudal aspect, but recent techniques have introduced plate application to the lateral and medial aspects. However, biomechanical rigidity comparisons between these methods have not yet been conducted. Elbow arthrodesis involves difficulty in plate contouring. In this study, a custom plate model was designed, and 10 models were classified on the basis of plate position, plate length, and the presence of additional fixation to the radius. Finite element analysis was used to compare the rigidity of each model.

**Materials and methods:**

A custom plate model was designed, and 10 finite element models were created based on CT data of a canine elbow. Models were categorized by plate position (caudal, medial, lateral), plate length (short vs. long), and the presence of additional radius fixation. An axial force of 150 N was applied to simulate loading, and peak von Mises stress and strain in the plate and bones (humerus, radius, ulna) were measured and compared across models.

**Results:**

Medial plate application demonstrated the highest rigidity in the plate, followed by lateral and then caudal application. In bone evaluation, the humerus and ulna showed greater rigidity with medial application. Rigidity of both plate and bone models increased with longer plate length and with additional fixation to the radius. For the radius, lateral fixation provided the greatest rigidity among groups with radius fixation.

**Discussion:**

Finite element analysis suggests that medial plate application provides superior biomechanical rigidity in canine elbow arthrodesis. Furthermore, utilizing a longer plate and incorporating additional fixation to the radius can enhance the overall biomechanical rigidity of the construct.

## 1 Introduction

Elbow arthrodesis is often chosen in canine patients when the joint becomes chronically painful and no longer supports functional use of the limb. This condition frequently results from advanced degenerative joint disease, failed total elbow replacement, persistent luxation or subluxation, complex intra-articular fractures that cannot be reconstructed, or peripheral nerve injury affecting limb function (1–4). The main goal of this procedure is to eliminate joint movement in order to achieve a stable and pain-free limb capable of bearing weight. Various fixation systems have been reported for canine elbow arthrodesis, including plates, external skeletal fixation, and hybrid constructs (1–4).

Traditionally, fixation plates have been placed on the caudal surfaces of the humerus and ulna (3, 5, 6). This method, however, involves an additional osteotomy of the ulna, which not only increases surgical complexity but also makes it more difficult to control angular and rotational alignment during the operation (1, 4, 6). To reduce these complications, alternative methods using plate application on the medial or lateral sides have been developed. In addition to medial application, lateral-only plate placement has also been reported in clinical cases of canine elbow arthrodesis (3). These approaches avoid osteotomy and allow easier access, though they are not without their own risks (4). Despite their increasing use, the relative biomechanical stability of these different approaches has not yet been thoroughly compared.

A clear understanding of how these surgical approaches perform mechanically is important when selecting the most effective fixation method. Biomechanical testing offers a way to assess how both surgical techniques and implants behave when subjected to loading. Several studies have used this approach to compare the rigidity of different constructs under various controlled conditions (7–11). That said, cadaver-based research presents several limitations. Issues such as ethical constraints, anatomical differences between individual specimens, and limited sample availability all limit the applicability of the findings to wider populations.

Finite element analysis (FEA) offers a useful alternative. This computer-based method divides complex anatomical structures into small units and calculates how stress, strain, and displacement are distributed across them. It enables controlled testing under consistent conditions while eliminating ethical and practical issues tied to cadaver use (9, 11, 12). As a result, FEA has been increasingly adopted in veterinary orthopedics, especially in the design and evaluation of surgical implants (9, 11–14).

Surgical plates that are tailored to specific procedures can offer several advantages. These include improved rigidity, shorter operative time, and better outcomes after surgery. Pre-contoured plates are already available for carpal and tarsal fusions, and this concept has been extended to the elbow as well. When plates are shaped in advance to match the dog's anatomy, the surgeon can avoid using goniometry during the procedure, especially for medial application (4). The development of 3D printing now allows for the production of highly customized implants, adapted to the individual and the surgical plan, which further improves mechanical performance and efficiency (15–17).

In this study, we explored the biomechanical rigidity of different plate placements in canine elbow arthrodesis, including medial, lateral, and caudal positions. We also evaluated how increasing plate length and adding fixation to the radius, in addition to the humerus and ulna, could affect overall rigidity. Our hypothesis was that medial and lateral placements would offer greater biomechanical advantage than caudal positioning. We also expected that longer plates and additional radius fixation would further enhance construct rigidity.

## 2 Materials and methods

### 2.1 Ethics statement

This study analyzed anonymized, retrospective computed tomography (CT) data that were acquired independently of this research; no prospective procedures on animals were performed. According to the institutional policy of Jeonbuk National University, analyses of de-identified retrospective imaging data do not constitute animal experimentation and are exempt from IACUC review. No owner-identifiable information was accessed.

### 2.2 Geometric model acquisition and reconstruction

A three-dimensional model of the right canine elbow joint was developed to perform FEA. The geometric dataset was sourced from archived CT scans at Jeonbuk Animal Medical Center (JAMC); these data were unrelated to the present study. The scans were derived from a 10.2 kg mixed-breed dog with no observable abnormalities in the forelimb. Imaging was conducted using an Alexion TSX-034A CT scanner (Canon Medical Systems, Tokyo, Japan) and the slice thickness was set to 0.5 mm. The resulting DICOM-format files were used for further modeling.

#### 2.2.1 Model groups definition

In this study, we created 10 plate configurations, which were grouped based on three main factors: the site where the plate was applied, the overall length of the plate, and the bones that were included in the fixation. The first classification was by application site. Plates were placed on the medial, lateral, or caudal surfaces of the limb, and each was labeled as Med, Lat, or Cau, respectively. Next, two different lengths were modeled for each application site. The short version, marked as S, covered about 50 percent of the length of the bone. This was the minimum length needed to allow the insertion of four screws into the humerus. The same proportion−50 percent—was also applied when modeling the plates for the ulna and radius. The longer configuration, labeled L, was designed to extend over roughly 70 percent of the bone's length in each case. In all models, screws were inserted into all available plate holes, and no empty holes were included. For short plate configurations, four screws were placed in the humerus and six screws in the ulna; when additional radius fixation was applied, four screws were inserted into the radius. For long plate configurations, seven screws were placed in the humerus and 10 screws in the ulna; when radius fixation was included, an additional eight screws were inserted into the radius.

In addition, for the medial and lateral applications, an extra set of models was developed to allow additional fixation to the radius along with the standard humeral and ulnar fixation (Supplementary Figure S1). These configurations were designated with an “R” label to indicate radius fixation. The R-models were also divided into short (R_Med_S, R_Lat_S) and long (R_Med_L, R_Lat_L) variants.

Although the caudal application group included fixation of both the ulna and radius, the term “R” was not applied in labeling. Nonetheless, data from the radius in these models were still collected and analyzed.

As a result, the 10 experimental groups were named: Med_S, Med_L, R_Med_S, R_Med_L, Lat_S, Lat_L, R_Lat_S, R_Lat_L, Cau_S, and Cau_L. A summary of the plate configuration groups is presented in Table 1.

**Table 1:** Classification of the 10 groups by plate application position, plate length, and additional radius fixation.

**Group ID**	**Plate position**	**Plate length**	**Radius fixation**
Lat_S	Lateral	Short	No
Lat_L	Lateral	Long	No
R_Lat_S	Lateral	Short	Yes
R_Lat_L	Lateral	Long	Yes
Med_S	Medial	Short	No
Med_L	Medial	Long	No
R_Med_S	Medial	Short	Yes
R_Med_L	Medial	Long	Yes
Cau_S	Caudal	Short	Yes
Cau_L	Caudal	Long	Yes

#### 2.2.2 Bone

CT data in DICOM format were first imported into Mimics 21.0 (Materialise, Leuven, Belgium). Using thresholding and segmentation tools, we isolated the humerus, radius, and ulna. A threshold of 500 Hounsfield Units (HU) was used to extract the cortical bone regions.

After segmentation, each bone mask was converted into an initial STL surface model. To improve anatomical clarity, we refined these models by applying manual labeling and smoothing to reduce artifacts and surface irregularities.

The refined STL models were next imported into Fusion 360 (Autodesk, California, USA). There, each model was manually repositioned so that its orientation matched the origin of the global coordinate system. To replicate a realistic surgical outcome, the elbow joint was adjusted to a fixed 130-degree angle, following values frequently cited in prior elbow arthrodesis studies (1–6). For consistency across all simulated models, the scapula was simplified. Only its distal portion was retained, ensuring uniform contact geometry during loading simulations.

#### 2.2.3 Implant

Implant models were created, including both plates and screws, with each plate designed to fit the anatomical configuration of its assigned group. The design process assumed the use of 3D printing as the fabrication method.

To approximate clinical practice, we modeled the implants as locking constructs, drawing on systems used in canine elbow arthrodesis. For the purpose of computational efficiency, thread details were not included. This approach is consistent with earlier finite element research involving locking plate systems (11, 16).

During the FEA setup, we specified contact definitions that could reproduce the mechanical interaction between the screw heads and the plate, thereby preserving the essential locking behavior.

**Figure 1:**
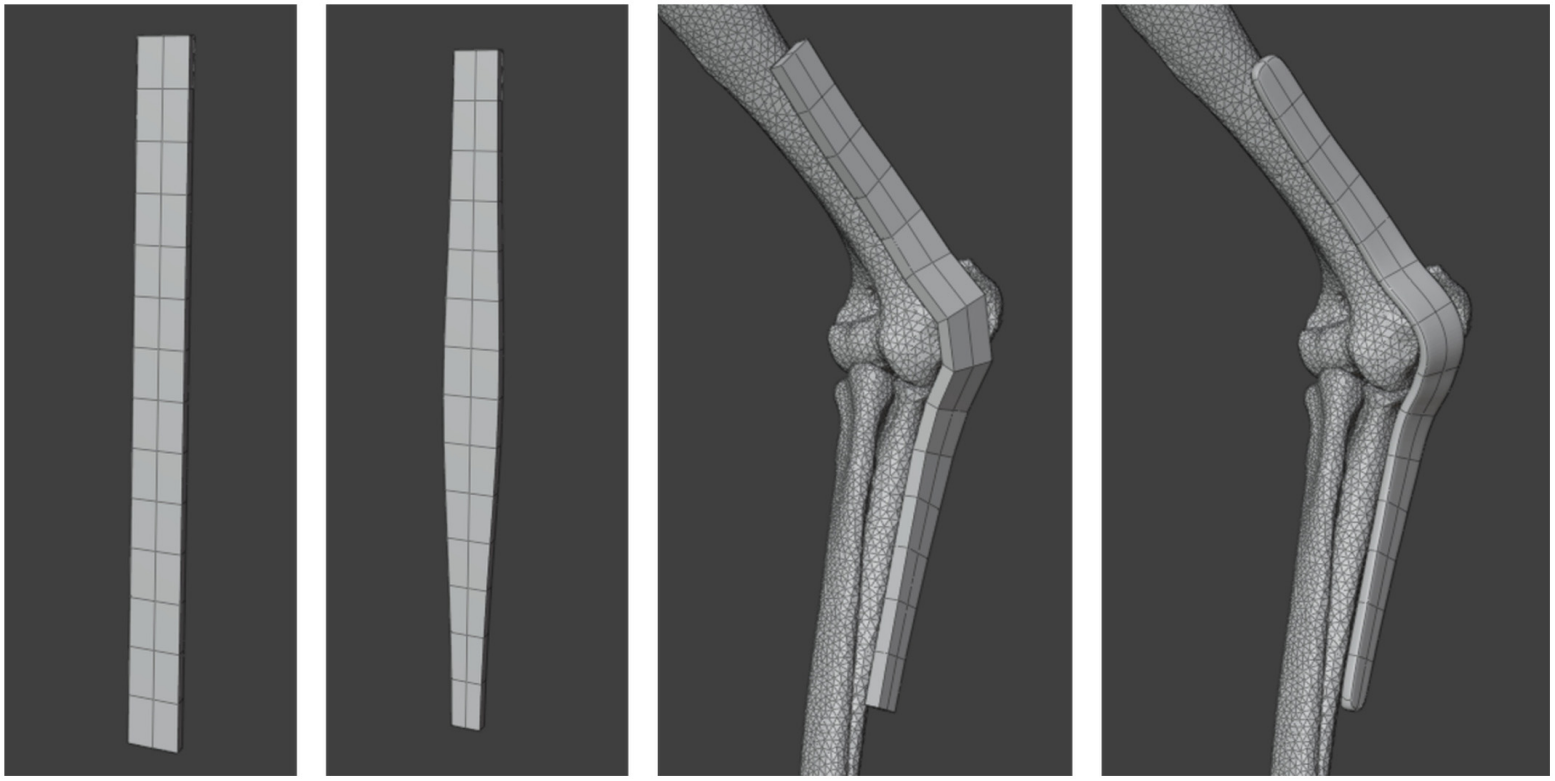
The plate model, initially created in Blender with a 10 mm width and 3 mm thickness, was subsequently modified to thicknesses of 6.5 mm distally and 12 mm over the central joint region, then contoured to maintain a gap of less than 2 mm relative to the bone model.

The plate model was created as a smooth, unthreaded structure. Implant modeling was carried out using a combination of Fusion 360 and Blender (Blender Foundation, Amsterdam, Netherlands), a mesh-editing and 3D modeling tool. The plate was initially modeled with a width of 10 mm and a constant thickness of 3 mm, which was later modified to reflect surgical realism: the distal portion was thickened to 6.5 mm, and the central joint region was reinforced to 12 mm thickness. The contour of the plate was digitally modified to keep its distance from the bone model surface below 2 mm (Figure 1).

To further streamline simulation, screw models were simplified as cylindrical shafts without threads, and their dimensions were adjusted relative to the local bone diameter. Specifically, 3.5 mm screws were modeled for the humerus, 2.7 mm screws for the ulna, and 2.0 mm screws for the radius, with all diameters restricted to no more than 40% of the bone diameter at the point of insertion. Screw holes were arranged with a uniform center-to-center spacing of approximately 10 mm along the plate length. The distance from each screw axis to the adjacent cortical bone edge was determined relative to local bone diameter and maintained within clinically acceptable margins to avoid cortical breach.

### 2.3 Finite element model generation

The geometric models comprising the bones, implant structures, and a simplified scapula were processed using external software tools to prepare for meshing. Once meshing was completed, all components were imported into FEBio Studio 2.8 (Musculoskeletal Research Laboratories, University of Utah, USA) for finite element analysis setup and simulation.

#### 2.3.1 Meshing

**Figure 2:**
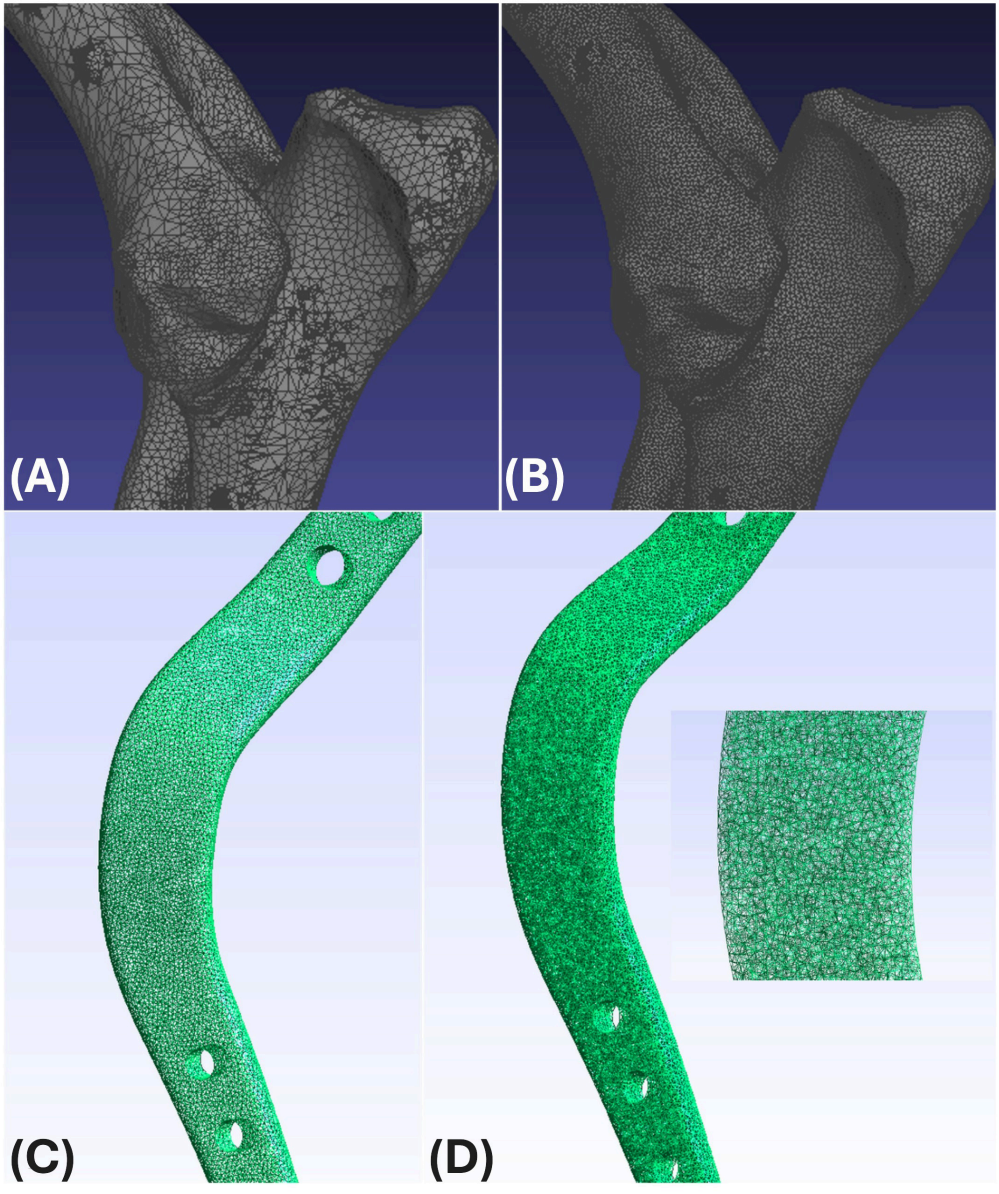
**(A)** The bone model source STL file. **(B)** After remeshing, the bone model. **(C)** Surface mesh. **(D)** Volume mesh.

After positioning the bone and implant models as required, we exported each component separately in STL format to preserve its original surface structure. The files were then imported into MeshLab 2023.12 (Visual Computing Lab, ISTI-CNR, Pisa, Italy), where remeshing was performed. In this stage, the surface was re-meshed using triangular elements with an average edge length of 0.5 mm. This step helped standardize resolution across models and contributed to consistent mesh quality (Figures 2A, B).

Once surface refinement was completed, the remeshed files were passed to Gmsh 4.11 (Geuzaine and Remacle, University of Liège, Belgium). There, each surface mesh was converted into a volumetric mesh composed of four-node linear tetrahedral elements (C3D4), which are commonly used in Finite Element Analysis (FEA) due to their computational efficiency and compatibility with complex geometries (Figures 2C, D).

The resulting volume meshes were then loaded into FEBio Studio for further processing. At this stage, each mesh was reviewed to verify that it was free of errors and suitable for simulation. After confirming the structural integrity and completeness of the models, they were prepared for the next steps involving the assignment of material properties and boundary conditions necessary for biomechanical evaluation.

#### 2.3.2 Material properties

Each material in the model was treated as having linear elasticity and being both homogeneous and isotropic (11–13). Material properties were primarily set based on previous studies; where canine-specific data was unavailable, data from humans or other animal models were referenced.

For implants, the plate and screws were assigned properties representing medical-grade titanium alloy (Ti-6Al-4V), with a density (ρ) of 4,500 kg/m^3^, a Poisson's ratio (ν) of 0.3, and a Young's Modulus (*E*) of 110 GPa (12). Bone properties were assigned based on literature values for cortical bone, which was defined with ρ = 2,000 kg/m^3^, *E* = 19 GPa, and ν = 0.3 (12, 13). The collateral, annular, and interosseous ligaments were positioned according to anatomically appropriate origin and insertion sites based on canine elbow anatomy. They were not modeled as three-dimensional anatomical structures; instead, linear discrete spring elements were used to reproduce their mechanical stabilizing function (18). Based on literature data for ligament stiffness, the medial/lateral collateral and annular ligaments were assigned a stiffness of 50 N/mm, and the interosseous ligament was assigned a stiffness of 100 N/mm (13, 19).

#### 2.3.3 Interface conditions

Each model was imported into FEBio (Musculoskeletal Research Laboratories, University of Utah, USA), where we defined contact interactions to simulate mechanical loading after implant fixation. For the interface between the screw head and the plate, we applied the tied-elastic contact option, as previously used to represent mechanical locking in similar finite element studies (11, 16).

For contact between bony surfaces, we used a sliding-elastic interface and set the friction coefficient to 0.121, referencing values from prior reports (20). We implemented the penalty method to handle contact enforcement, and the initial penalty value was based on each material's Young's modulus. To improve solver performance, we also enabled the auto-penalty setting, which dynamically updated the value during simulations for better convergence control (21).

#### 2.3.4 Boundary conditions

To constrain the model appropriately, the distal surfaces of the radius and ulna were fully fixed in all translational degrees of freedom (*x* = 0, *y* = 0, *z* = 0), thereby simulating rigid fixation against the ground (22). A standing posture was assumed for all simulations, with the global coordinate system defined such that the XY-plane represented the ground plane and the *Z*-axis represented the vertical direction. The elbow joint was fixed at 130° of elbow flexion. To ensure that the applied force was directed primarily along the *Z*-axis, the simplified scapula model, which articulates with the humeral head, was restricted from translating in the X and Y directions (*x* = 0, *y* = 0), while free translation along the *Z*-axis and full rotational freedom were permitted (Supplementary Figure S2).

#### 2.3.5 Loading conditions

To simulate loading that reflects real physiological conditions, we applied a vertical force along the *Z*-axis through the simplified scapula. Trotting has been reported to generate forelimb ground reaction forces up to 1.1 times body weight in dogs. To model a more challenging scenario, the load was increased to 1.5 times body weight. Based on the subject's body mass, this value corresponded to 150 N (23, 24).

### 2.4 Simulation and data analysis

We performed finite element simulations using FEBio 4.8 (Musculoskeletal Research Laboratories, University of Utah, USA) as part of this study. All models were analyzed under the same boundary conditions and solver parameters to ensure consistency and allow direct comparison between groups.

For each simulation, we recorded the peak von Mises stress and von Mises strain values. These measurements were used to evaluate the biomechanical rigidity of each configuration and to identify areas of highest stress or strain concentration. Only static axial compression was analyzed in this study; no cyclic loading, fatigue analysis, or target deformation criteria were assumed.

## 3 Results

### 3.1 Implant model results

**Figure 3:**
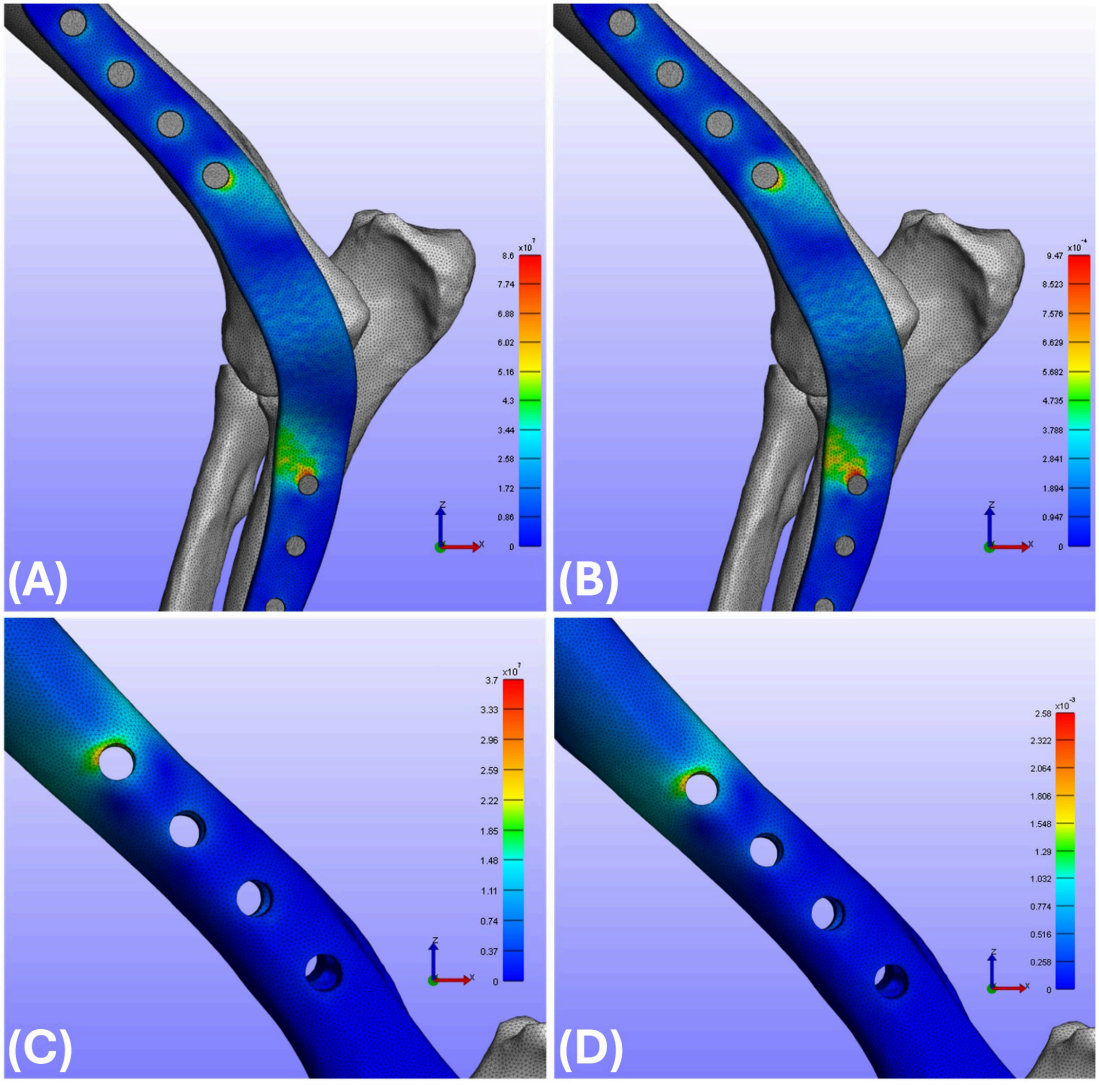
**(A)** von Mises stress results for Med_S group plates. **(B)** von Mises strain results for Med_S group plates. **(C)** von Mises stress results for Med_S group humerus. **(D)** von Mises strain results for Med_S group humerus. Med, Medial; S, Short.

**Figure 4:**
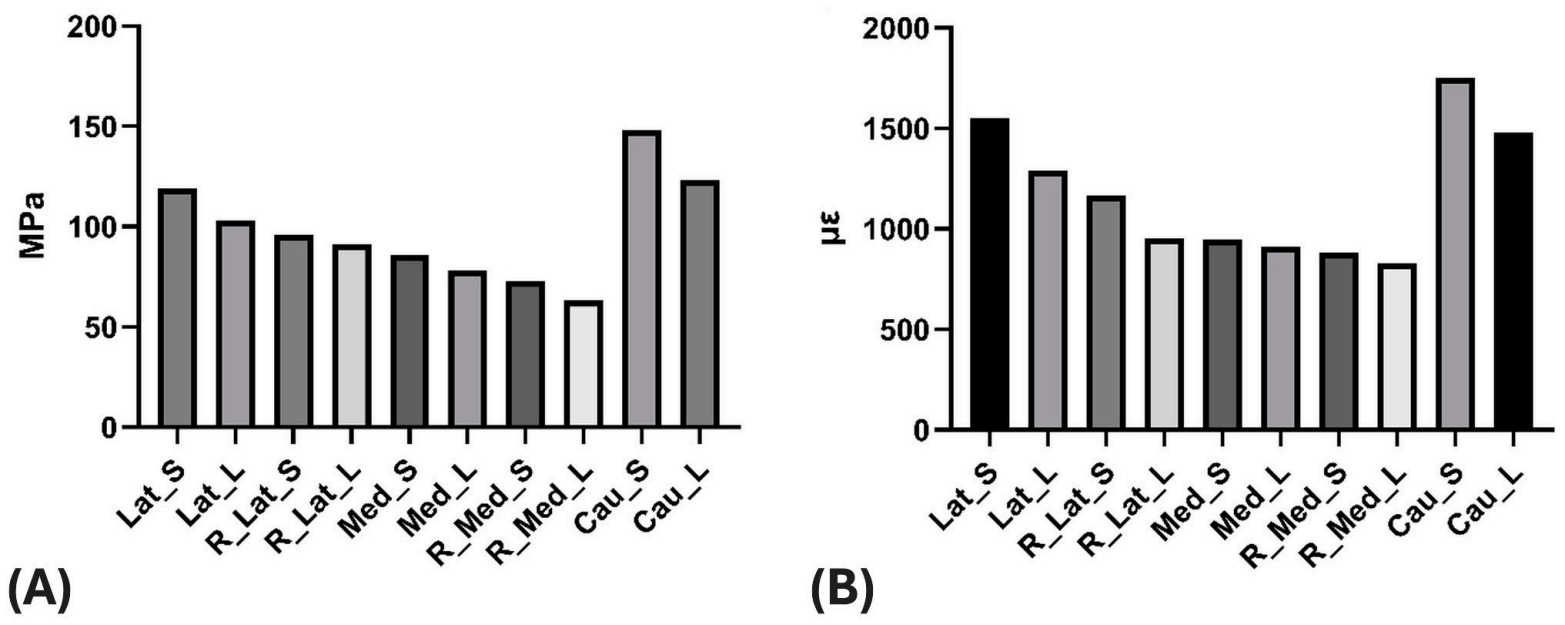
**(A)** Maximum von Mises stress in the plate per group. **(B)** Maximum von Mises strain in the plate per group. Lat, Lateral; Med, Medial; Cau, Caudal; S, Short; L, Long; R, Radius fixation.

**Figure 5:**
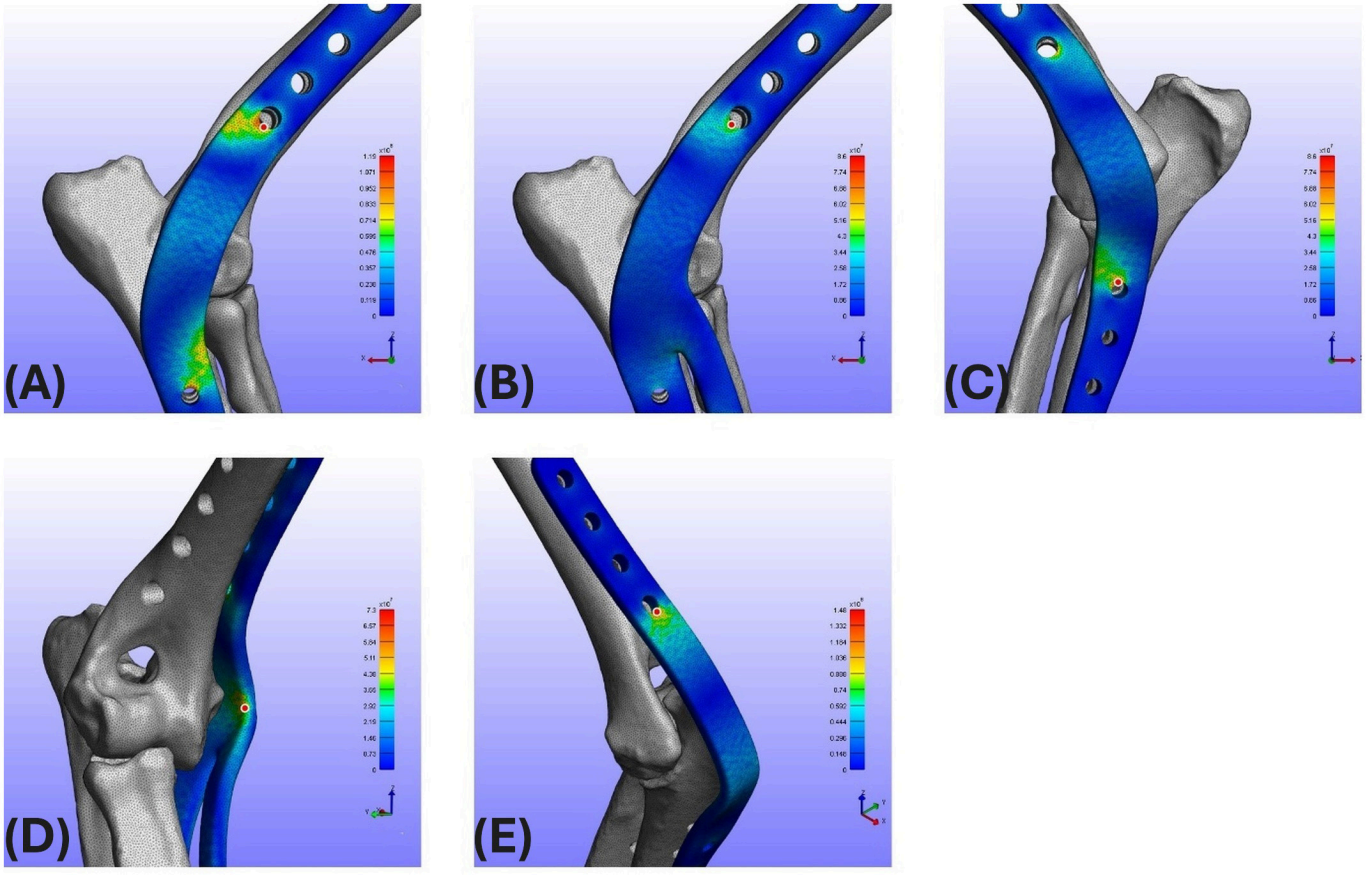
Locations of maximum von Mises stress concentration. **(A)** Lat group: identified at the most distal aspect of the humeral region. **(B)** R_Lat group: identified at the most distal aspect of the humeral region. **(C)** Med group: identified at the proximal aspect of the ulnar region. **(D)** R_Med group: identified at the cranial aspect of the elbow joint region. **(E)** Cau group: identified at the most distal aspect of the humeral region. Lat, Lateral; Med, Medial; Cau, Caudal; R, Radius fixation.

Most experimental groups demonstrated consistent correspondence between von Mises stress and strain distributions, with similar spatial patterns observed across configurations (Figure 3). The maximum von Mises stress and strain values observed on the plate for each of the 10 experimental groups are listed in Tables 2, 3, respectively. Regarding maximum von Mises stress, the caudal groups exhibited values of 148 and 123 MPa for S and L lengths, respectively. The lateral groups showed values of 119 MPa (S) and 103 MPa (L), while the medial groups showed 86 MPa (S) and 78 MPa (L). For maximum von Mises strain, the caudal groups exhibited 1,751 and 1,481 με for S and L, the lateral groups showed 1,552 (S) and 1,063 με (L), and the medial groups showed 947 (S) and 910 με (L). Comparison between the medial, lateral, and caudal application groups revealed that higher von Mises stress and von Mises strain were observed in the order of caudal, lateral, and medial groups (Figure 4). The effect of plate length was also investigated. Comparing the short (S) and long (L) plate models within each application site showed that, overall, longer plates exhibited lower von Mises stress and von Mises strain, with short plates demonstrating approximately 9%−17% higher stress and 4%−32% higher strain compared with long plates, depending on application site. For the R groups in the medial and lateral positions (where the radius was additionally fixed), the maximum von Mises stress was 96 (S) and 91 MPa (L) for R_Lat, and 73 (S) and 63 MPa (L) for R_Med. The corresponding maximum von Mises strains were 1,552 (S) and 1,063 με (L) for R_Lat, and 883 (S) and 831 με (L) for R_Med. Groups with additional radius fixation showed lower von Mises stress and von Mises strain compared to groups fixing only the ulna and humerus, with reductions of approximately 12%−19% for stress and 7%−25% for strain. The location of the peak stress/strain on the plate varied depending on the plate application site and the addition of radius fixation, independent of plate length. Specifically, for lateral (both with and without radius fixation) and caudal applications, the maximum point was located at the most distal aspect of the humeral portion (Figures 5A–C). For medial application, without radius fixation, the maximum point was near the proximal aspect of the ulnar portion (Figure 5C). When radius fixation was added medially, the maximum point shifted to the cranial aspect near the elbow joint region (Figure 5D). For the caudal application group, the maximum von Mises stress concentration was identified at the most distal aspect of the humeral region (Figure 5E).

**Table 2:** von Mises stress values for the implant and bone models (Unit: MPa).

**Group ID**	**Plate**	**Humerus**	**Ulna**	**Radius**
Lat_S	119	43	45	–
Lat_L	103	32	31	–
R_Lat_S	96	40	17	19
R_Lat_L	91	30	16	12
Med_S	86	37	33	–
Med_L	78	20	21	–
R_Med_S	73	41	16	27
R_Med_L	63	18	13	22
Cau_S	148	39	23	34
Cau_L	123	21	21	26

**Table 3:** von Mises strain values for the implant and bone models (Unit: microstrain, με).

**Group ID**	**Plate**	**Humerus**	**Ulna**	**Radius**
Lat_S	1,552	2,942	3,106	–
Lat_L	1,063	2,139	2,167	–
R_Lat_S	1,165	2,770	1,242	1,501
R_Lat_L	952	1,865	1,113	854
Med_S	947	2,580	2,791	–
Med_L	910	1,858	1,754	–
R_Med_S	883	2,843	1,140	1,853
R_Med_L	831	1,334	919	1,447
Cau_S	1,751	2,697	1,582	2,461
Cau_L	1,481	1,486	1,483	1,758

### 3.2 Bone model results

Maximum von Mises stress and strain for the bone components (humerus, ulna, and radius) within each of the 10 experimental groups are listed in Tables 2, 3, respectively. A general observation across all plating sites was that employing longer plates consistently resulted in lower stress and strain levels within the bone components.

**Figure 6:**
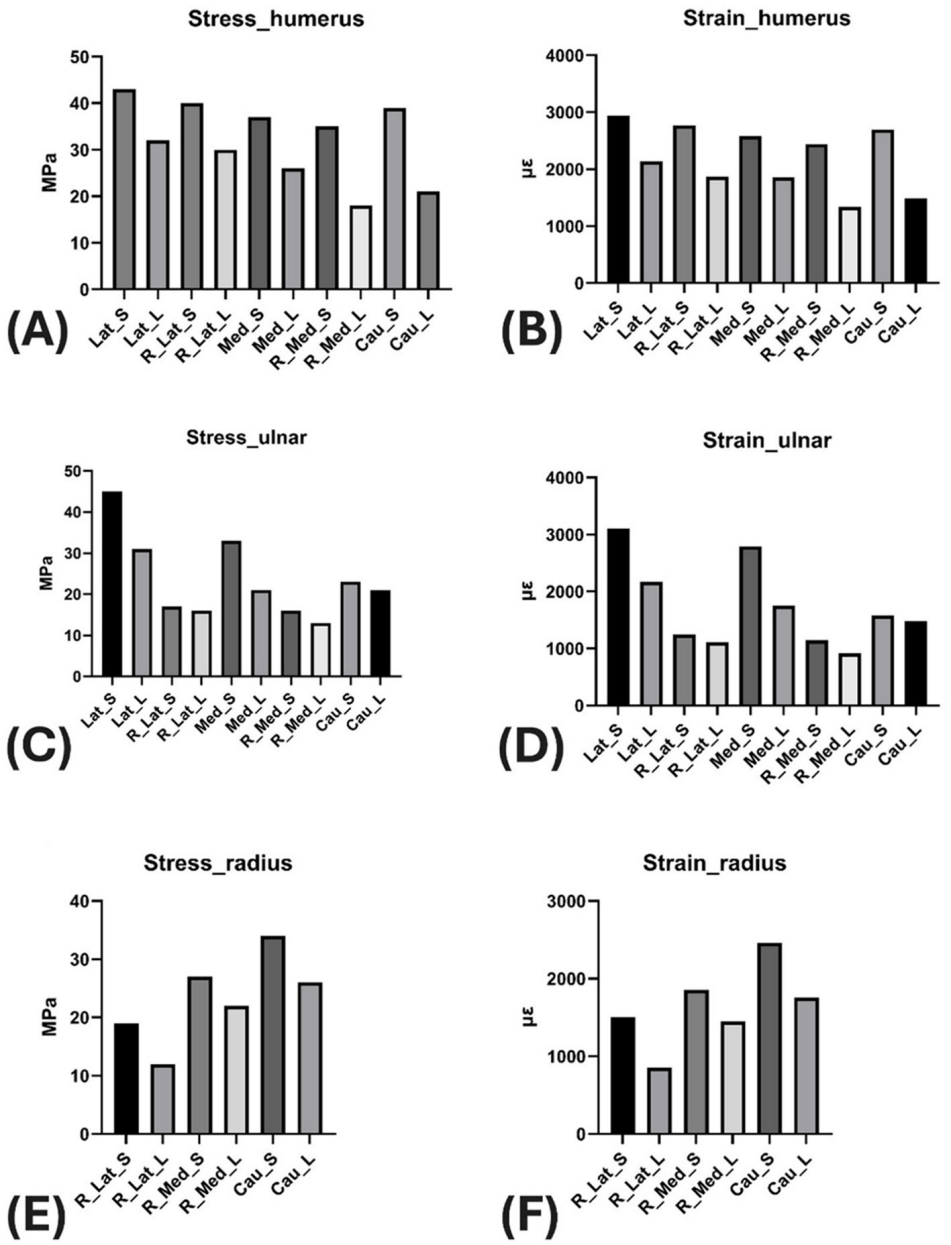
**(A)** Group-wise maximum von Mises stress in the humerus. **(B)** Group-wise maximum von Mises strain in the humerus. **(C)** Group-wise maximum von Mises stress in the ulna. **(D)** Group-wise maximum von Mises strain in the ulna. **(E)** Group-wise maximum von Mises stress in the radius. **(F)** Group-wise maximum von Mises strain in the radius.

Focusing on the humerus, the shorter plate groups yielded generally similar, elevated maximum von Mises stress and strain (Figures 6A, B). The effect of incorporating additional radius fixation into the construct was minimal on the humeral stress state. When evaluating the long plate groups, lateral application was associated with higher stress and strain in the humerus compared to the medial and caudal application groups, which showed comparable outcomes relative to each other.

Turning to the ulna, the analysis indicated that combining longer plates with supplementary radius fixation generally led to a reduction in maximum von Mises stress and strain (Figures 6C, D). The results for the ulna were similar between the groups that included radius fixation (R_Med and R_Lat groups). For groups without radius fixation, lateral plate application (Lat_S, Lat_L) was associated with higher ulnar stress and strain compared to medial application (Med_S, Med_L) (Figures 6E, F).

An observation specific to the ulna was that longer plate lengths appeared associated with lower maximum von Mises stress and strain. Comparing application sites for the ulna, caudal plating yielded higher stress and strain levels, while lateral plating resulted in lower magnitudes.

## 4 Discussion

This study utilized finite element analysis (FEA) to comparatively analyze the biomechanical rigidity according to plate application site (caudal, medial, lateral) in canine elbow arthrodesis. A total of 10 groups were modeled with plate length and additional radius fixation as variables, and the maximum von Mises stress and strain in the plate and bone components of each model were compared to relatively evaluate biomechanical rigidity.

The main findings can be summarized into three points: (1) In terms of the plate's own rigidity, medial application was the most advantageous (Medial > Lateral > Caudal); (2) Construct rigidity improved as the plate length increased (longer length); and (3) Rigidity was also enhanced when additional radius fixation was performed. In the case of bone components, somewhat different results were observed depending on the site (humerus, ulna, radius) and model conditions, but overall, medial application, long plates, and radius fixation showed favorable results in the humerus and ulna as well. For the radius, within the groups where radius fixation was performed, the order of rigidity was Lateral > Medial > Caudal. Comprehensively, this suggests that fixation including the radius with a sufficiently long plate via a medial approach is the most biomechanically stable method.

The superior biomechanical performance of medial plate application can be explained by load transfer mechanisms and bending behavior of long bones. During weight-bearing, axial loading across the elbow arthrodesis construct generates bending moments that produce tension on one surface and compression on the opposite surface. Plates positioned closer to the effective tension surface resist bending more efficiently and experience lower stress and strain compared to plates placed on the compression surface (4, 13).

In this context, medial plate application provides a more favorable alignment with physiological load transfer pathways than lateral or caudal placement. In contrast, caudal application is more susceptible to bending and edge loading, which may contribute to increased stress and strain concentrations observed in the present study.

Considering the axial load transfer mechanism during weight-bearing on the canine forelimb, as suggested in previous studies (4, 13), medial and lateral plate application can theoretically be biomechanically more stable than caudal application due to edge loading. The results of this study also support this theory, showing lower plate stress and strain values in the medial and lateral groups compared to the caudal group. The result showing higher rigidity for medial application compared to lateral application can be explained by the difference in required plate contouring. Applying the plate laterally requires more contouring than applying it medially, and this result is consistent with findings from previous research showing that stress increases with the degree of plate contouring (4, 8, 25, 26). Therefore, the medial approach, requiring relatively less contouring, is thought to possess higher biomechanical rigidity.

This influence of contouring can also be considered in relation to the location where maximum von Mises stress and strain were observed on the plate (location of peak stress/strain). Due to the characteristics of the locking screw system, load transfer between the plate and bone occurs primarily through the screws, so stress generally tends to concentrate around the screw closest to the joint. Although most of our observations aligned with expected trends, a clear difference became apparent in the R_Med designated groups (R_Med_S and R_Med_L). For these particular groups, peak stress and strain were found near the cranial (head-side) portion of the implanted plate, especially in the area around the joint. A likely reason for this is that the medial surgical approach required more significant shaping of the plate to securely fix it to the radius; this shaping process might have, as a consequence, led to a localized concentration of stress.

To examine whether incorporating radius fixation offers biomechanical benefits, we included models featuring this design, although it is not commonly adopted in standard surgical routines. In both medial and lateral plate configurations, the inclusion of radius fixation led to a consistent reduction in stress and strain levels.

This effect was seen not only in the implant itself but also in adjacent bones such as the humerus and ulna. Based on these findings, extending fixation to the radius may improve the mechanical integrity of the construct compared to traditional two-bone approaches.

The trend of decreasing stress/strain in both the plate and bone as plate length increased was clearly observed. This result is consistent with previous studies (27), which reported that longer plates allow for more uniform stress distribution (promoting more uniform stress distribution) and increase construct stiffness, making them biomechanically more stable. Therefore, using the longest possible plate within the clinically applicable range is recommended.

This study has several inherent limitations based on FEA simulation. First, FEA does not perfectly simulate actual cadaveric tests or the *in vivo* environment. Although the validity of FEA has been demonstrated in many studies, it is difficult to definitively state that simulation results always align with actual clinical outcomes, and further experimental validation is needed. Second, the plate used was a simplified model created for research purposes, not an actual commercial product, and the radius additional fixation design, in particular, may require patient-specific 3D printing technology for actual clinical application. Third, for calculation and finite element analysis, the analysis assumed homogeneous and isotropic properties, which differ from the actual properties of bone. Although von Mises stress and strain were analyzed based on these properties, there is a difference from actual characteristics. Furthermore, the actual complex joint structure and its motion were partially simplified in the FEA modeling process. This is also a necessary process for finite element analysis and calculation, but differences from reality may exist. There were also limitations in model resolution and analysis accuracy, which were partly influenced by the level of available computing resources. Furthermore, it is important to highlight that the scope of the present investigation was restricted to examining solely one static axial compression scenario. Other types of loading, such as dynamic forces that occur during walking or running, or multi-directional stress experienced in real joints, were not included in this analysis.

## 5 Conclusion

FEA study can provide data indicating that medial plate application offers the highest biomechanical rigidity in canine elbow arthrodesis. It also strongly suggests that increasing plate length and adding radius fixation can further enhance biomechanical performance.

## Data Availability

The raw data supporting the conclusions of this article will be made available by the authors, without undue reservation.

## Supplementary material

SUPPLEMENTARY FIGURE S1 Three-dimensional illustration of the short medial plate configuration including additional radius fixation, showing the relationship between the plate, humerus, ulna, and radius in the corresponding models.

SUPPLEMENTARY FIGURE S2 Simplified scapula model used for load application. The model was designed as a rigid load-transmitting surrogate articulating with the humeral head and does not represent the anatomical geometry of the scapula. The applied load was transferred along the vertical *Z*-axis.
