# Supplementary figures and images for: Finite element analysis of plate placement in canine elbow arthrodesis: comparison of caudal, medial, and lateral applications
Source: Front Vet Sci. 2026 Jan 16;12:1705303. doi: 10.3389/fvets.2025.1705303 (PMC12855140; doi:10.3389/fvets.2025.1705303)

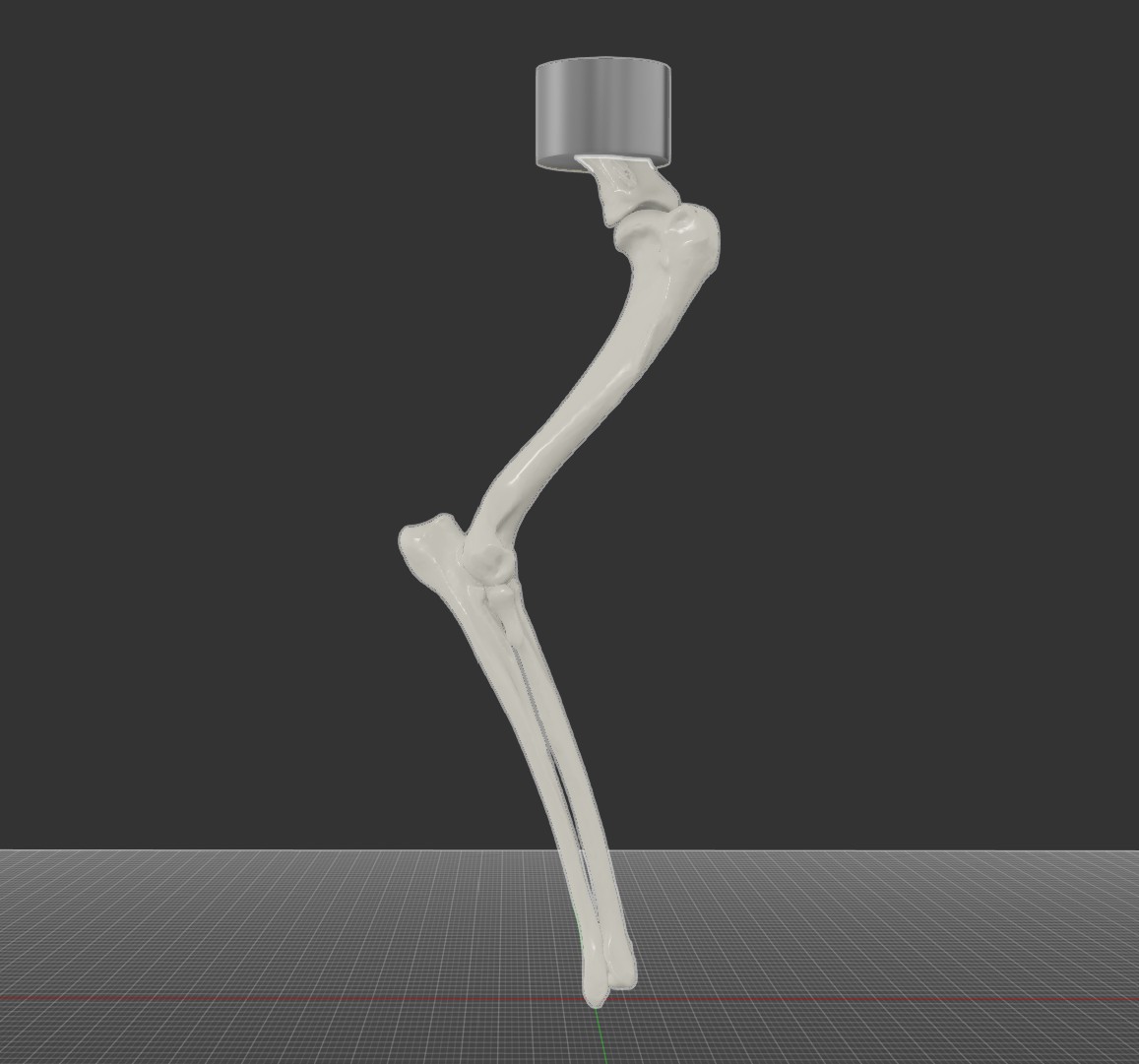

Supplement: SUPPLEMENTARY FIGURE S1 — Three-dimensional illustration of the short medial plate configuration including additional radius fixation, showing the relationship between the plate, humerus, ulna, and radius in the corresponding models. [file Image_1.jpeg]

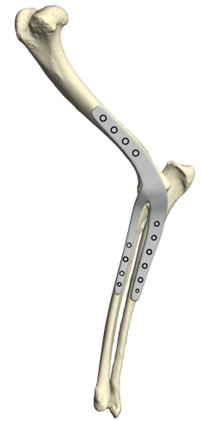

Supplement: SUPPLEMENTARY FIGURE S2 — Simplified scapula model used for load application. The model was designed as a rigid load-transmitting surrogate articulating with the humeral head and does not represent the anatomical geometry of the scapula. The applied load was transferred along the vertical Z-axis. [file Image_2.jpeg]
